# Validation of the Scandinavian neurotrauma committee guidelines – A retrospective study in region Örebro county

**DOI:** 10.1016/j.bas.2025.104231

**Published:** 2025-03-09

**Authors:** Samuel Jara Josefsson, Dhanisha Trivedi, Patrick Vigren, András Büki

**Affiliations:** a School of Medical Sciences, Faculty of Medicine and Health, Örebro University, Örebro, Sweden; b Department of Neurosurgery, Region Örebro County, Örebro University Hospital, Örebro, Sweden

**Keywords:** Adults, CT, Clinical guidelines, Traumatic brain injury, Validation

## Abstract

**Introduction:**

Traumatic Brain Injury (TBI) is a global health concern and a leading cause of trauma-related death worldwide. Computed tomography (CT) scan is the gold standard for screening for intracranial bleeding following TBI. Most cases of TBI are mild, with negative CT scans. Different instruments and guidelines are employed to better predict which patients need a CT scan and to minimise unnecessary radiation exposure and save resources. One such instrument is the Scandinavian Neurotrauma Committee guidelines.

**Research question:**

To validate and examine adherence to the Scandinavian Neurotrauma Committee guidelines in Region Örebro County.

**Material and methods:**

We executed a retrospective study with review of patient records and data analysis. Descriptive and comparative statistics were used, along with binary logistic regression analysis to account for confounding factors.

**Results:**

A total of 505 cases were reviewed. Sensitivity of the guidelines was measured at 95% with specificity at 29%. The positive and negative predictive values were 0.77 and 0.69, respectively. A total of 17 false negative cases were found. One case required surgery, during which a chronic subdural hematoma was identified. Adherence to guidelines was 56%, with the lack of analysis of S100B primarily accounting for non-adherence. A total of 54 CT scans were performed outside of guideline indications.

**Discussion and conclusions:**

The guidelines can effectively predict which patients need a CT scan. Increased adherence could potentially decrease the number of CT scans, while inclusion of older age limit as an independent rule-in law for CT scans would increase patient safety.

## Highlights

• The Scandinavian Neurotrauma Committee Guidelines is a sensitive screening tool.
• Adherence to guidelines in the emergency department can be improved.
• Improved adherence could lead to fewer CT scans being performed.
• Age as a sole rule-in criterion for CT scan should be considered in future revision.

## Abbreviations

ATLS: Advanced Trauma Life Support
S100B: Calcium-binding protein B
CCTHR: Canadian CT Head Rule
X^2^: Chi-squared test
CT: Computed Tomography
CI: Confidence Interval
CHIPPR: CT in Head Injury Patients Prediction Rule
GCS: Glascow Coma Scale
ICD: International Statistical Classification of Diseases and Related Health Problems
NICE: National Institute for Health and Care Excellence
NPV: Negative Predictive Value
NOHCTR: New Orleans Head CT Rule
PPV: Positive Predictive Value
RLS: Reaction Level Scale
RÖC: Region Örebro County
SNC: Scandinavian Neurotrauma Committee
TBI: Traumatic Brain Injury

## 1 Introduction

Traumatic brain injury (TBI) is a leading cause of trauma-related death and disability in Europe (Majdan et al., 2016). While severe TBI has long dominated international research on neurotrauma, mild and moderate TBI cases account for over 90% of head injuries (Dewan et al., 2019). Significant steps have been taken globally to reduce the incidence of TBI, such as policy changes, and helmet usage promotion, resulting in a somewhat decreased prevalence. However, despite these advancements, TBI still remains a global health concern with an incidence rate of at least 346 per 100 000 population (Guan et al., 2023; Sone et al., 2017).

Prompt identification and diagnosis of patients with TBI is essential in preventing outcome deterioration due to secondary injuries. The gold standard for the diagnosis and evaluation of TBI in the acute setting, is computed tomography (CT scan) (Waganekar et al., 2018; Toyama et al., 2005). In the Nordic countries, CT scan of the head is among the top 20 most common radiological examinations, leading to an official statement from the Nordic Radiation Protection cooperation, which expresses concerns about the increased use of CT scans in with an estimated 20% of all CT scans being deemed unnecessary (The Nordic Radiation Protection co-operation, 2012). Concerns are also being raised regarding the association between early-life radiation exposure and an increased incidence of brain tumors later in life, a link that research has found to be plausible. (Brenner and Hall Eric, 2007; Davis et al., 2011). The economic aspect of unnecessary CT scans has also added to the recent strain on the healthcare systems (Hendee et al., 2010).

Several strategies have therefore been developed, aiming to lower CT scan usage. These strategies focus primarily on clinical findings such as patient history and/or signs and symptoms upon clinical examination, which could stratify patients at risk of deterioration or needing interventions and thus would warrant a CT scan.

A comparison between the contemporary guidelines for CT scan decision-making in head injury patient cases (Scandinavian Neurotrauma Committee guidelines (SNC), CT in Head Injury Patients Prediction Rule (CHIPPR), New Orleans Head CT Rule (NOHCTR), Canadian CT Head Rule (CCTHR), and the National Institute for Health and Care Excellence (NICE) guidelines) is shown in Appendix A (Smits et al., 2007; Stiell et al., 2001; Haydel and Blaudeau, 2000; National Institute for Health and Care Excellence). Notably, while age is included in the SNC algorithm, it is not alone enough to warrant a CT scan, unlike in the other guidelines. Instead, age ≥65 in combination with anti-platelet medication is cause for CT scan, provided that the patient has symptoms of TBI. Furthermore, the SNC guidelines also omit dangerous mechanism of injury (i.e. pedestrian hit by car) as a criterion for CT scan.

In recent years, studies have been conducted on the possible use of biomarkers to predict the need for CT scan, both as standalones and as incremental to existing guidelines. Calcium-binding protein B (S100B) has proven to be a promising candidate (Czeiter et al., 2020; Rogan et al., 2023; Trivedi et al., 2024). The SNC guidelines have been pioneering the inclusion of biomarkers into existing triage algorithms, until recently. The updated French Guidelines have similarly implemented S100B levels to aid in determining the patients at risk of CT-positive TBI and a similar multidisciplinary Spanish protocol has also been presented recently (Gil-Jardiné et al., 2023; Ruiz et al., 2024). They have also included novel biomarkers such as glial fibrillary acidic protein (GFAP) and ubiquitin C-terminal hydrolase L1 (UCH-L1) and recommend the newly-FDA approved combined test for patients presenting up to 12 h after injury (Bazarian et al., 2018).

## 2 Aim

This study aimed to validate and examine the adherence to the SNC guidelines in Örebro county of Sweden, with the hypothesis being that the guidelines are sensitive in predicting pathological CT findings and that increased adherence to the guidelines results in fewer unnecessary CT scans performed.

## 3 Method

### 3.1 Sample

**Fig. 1:**
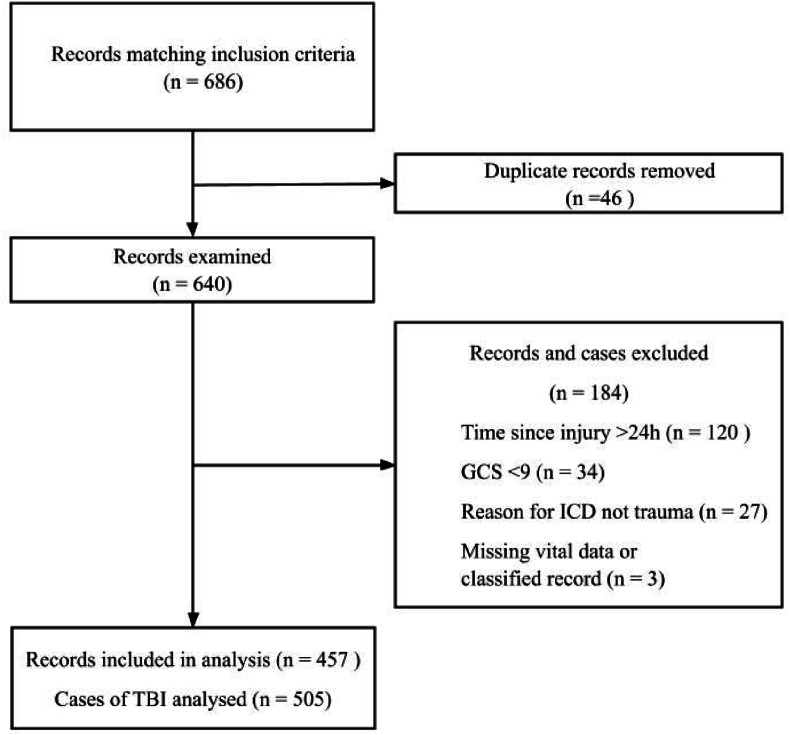
– Process of inclusion to analysis. GCS – Glascow coma scale, TBI – Traumatic brain injury, ICD – International classification of diseases and related health problems.

The sample was obtained from existing patient records in InfoMedix, the county digital patient administrative system. Patients matching the inclusion criteria were assigned a unique pseudonym which was then entered into the regional electronic records software Klinisk Portal, to access their medical records. The inclusion criteria were patients ≥18 years of age who were admitted to one of the three emergency departments in Region Örebro County (RÖC) (Örebro, Lindesberg, Karlskoga) between 2019 and 2023, and whose visit was coded with ICD-10 code S06 (intracranial injury). This generated 686 patient records, some of which were duplicates. Exclusion criteria were as follows: presentation to one of the above emergency rooms ≥24 h from injury, initial GCS <9, no head trauma, missing data regarding the key variables, or records that could not be accessed (classified). This resulted in 505 cases from 457 patient records (Fig. 1).

The following data were collected from the patient records: sex, age, hospital location, length of stay, time since injury, GCS/Reaction Level Scale (RLS) score, TBI symptoms, anticoagulant and/or antiplatelet medication use, and results from CT scans and S100B when available. The data was analysed on a case level rather than on individual patient records. RLS score is the most commonly used consciousness scale in Sweden, compared to GCS, in the pre-hospital setting as well as in the emergency department. To cohere with the SNC guidelines, using GCS for categorizing injury (moderate, medium, minimal), RLS scores were converted into GCS scores in cases where the patient records only mentioned the RLS. The conversion was made based on previous publications as follows: RLS 1 = GCS 15, RLS 2 = GCS 14, RLS 3 = GCS 13-9, RLS ≤4 = GCS ≤8 (Starmark et al., 1988; Undén et al., 2013).

### 3.2 Data analysis

All data were recorded in Excel® and later exported to IBM® SPSS® Statistics (version 28.0) for analysis. General descriptive methods were used to present the data, including mean, median, percentage, percentiles, and binning of data (such as age groups). Median with percentiles was used to present data that were not normally distributed. Tests of normality were done using both Shapiro-Wilk and Kolmogorov-Smirnov tests, as well as visually with Q-Q plots. The Chi-squared test (X^2^) was used to analyse binary data and compare binary, categorical, or ordinal data to determine association rather than independence, between variables. In all tests analysed with X^2^, zero cells had expected counts of less than five. When determining difference between binary and ordinal variables, or between means of non-normally distributed data, the Mann-Whitney *U* test was used instead. Association between variables was further examined using Spearman's rank correlation coefficient (Spearman's rho). All data analysed with this method was ordinal in nature and not normally distributed. Binary logistic regression analysis was used to determine coefficients between binary variables. Binary logistic regression was also used when analysing binary data while accounting for confounding factors. An example of such an analysis was adherence to guidelines (dependent variable), with sex, age, location, GCS at arrival, and SNC category as covariates.

Guidelines sensitivity was calculated by dividing the number of true positive cases by the total number of positive CT results. Specificity was calculated by dividing the total number of true negative cases by the total number of negative CT results. The positive predictive value (PPV) was calculated as the quotient of true positive CT results, by the total number of true and false positive results. The negative predictive value (NPV) was calculated as the quotient of true negative CT scans, by the total number of true and false negative results. The confidence interval of 95% was calculated using the Wilson score interval. Throughout the analysis, p < 0.05 was considered statistically significant.

### 3.3 Ethical considerations

The study was conducted in accordance with the ethical principles of the latest version of the Declaration of Helsinki (World Medical Association, 2013). Originally, the data was gathered as part of a quality assurance project, in collaboration with the Department of Neurosurgery at Örebro University Hospital and with approval from the head of the department. All relevant documentation has been signed and electronically stored (Dnr: 24RS711). The project was granted ethical approval from the Swedish Ethical Review Authority (Dnr: 2024-02330-01).

## 4 Results

This study included 505 cases of TBI. The demographics of the sample are outlined in Table 1. Men were more likely to have more severe TBI as characterised by the SNC guidelines and more likely to be admitted for in-hospital observation and/or treatment. Correlation analysis showed coefficients of −0.101 and 0.091, respectively (p < 0.05 in both cases). Women had higher GCS scores on average at presentation. Age was not normally distributed but did not differ between sexes. Increased incidence of TBI was observed at two age peaks: in the age group 31–40 years old and then again at 71–90 years old. A total of 93% of cases presented with GCS 14–15, which correlates to minimal and mild TBI, according to SNC classifications.

**Table 1:** Characteristics of the sample presented on sex and summarised in total.

		Female	Male	Total (%)	p-value
**n (%)**		209 (41)	296 (59)	505	
**Age median (q1/q3)**		77 (54/85)^1^	67 (55/82)^1^	74 (55/84)^1^	0.111
**Age group**	<30	28	27	55 (11)	0.189
	31–40	12	16	28 (28)	
	41–50	6	18	24 (5)	
	51–60	19	28	47 (9)	
	61–70	15	47	62 (12)	
	71–80	46	69	115 (23)	
	81–90	65	75	140 (28)	
	>90	18	16	34 (7)	
**Hospital**	Örebro	93	126	219 (43)	0.499
	Lindesberg	54	68	122 (24)	
	Karlskoga	62	102	164 (32)	
**SNC Category**	Minimal	44	45	89 (18)	0.041^a^
	Mild-Low	73	98	171 (34)	
	Mild-Medium	20	42	62 (12)	
	Mild-High	65	81	146 (29)	
	Moderate	7	30	37 (7)	
**GCS on arrival**	9	1	5	6 (1)	0.023∗
	10	1	4	5 (1)	
	11	1	5	6 (1)	
	12	1	5	6 (1)	
	13	3	12	15 (3)	
GCS on arrival (cont)	14	36	53	89 (18)	
	15	166	212	378 (75)	

Numbers in parentheses mark percentage of the total unless otherwise specified. p-value for the difference between sexes in the categories as a whole.

a Statistical significance (p < 0.05),^1^ 1st/3rd quartile. SNC – Scandinavian Neurotrauma Committee, GCS – Glasgow Coma Scale.

A total of 453 CT scans were performed, and 325 of these (72%) revealed pathological findings. According to the SNC guidelines, CT scans were recommended in 416 of these cases. S100B were not analysed in 124 cases, even though it was recommended by the SNC guidelines. Table 2 shows the relationship between recommendation and result, further elaborating that the sensitivity of the SNC guidelines in this study was 95% (95% CI, 92–97%). The specificity was 29% (95% CI, 22–37%). The PPV and NPV in this study were 0.77 (95% CI, 0.73–0.81) and 0.69 (95% CI, 0.55–0.79), respectively.

**Table 2:** Number of cases where SNC recommended CT scan as compared to whether or not a CT scan was performed and its result.

		n (%)	CT performed		CT pathological	
			No (%)	Yes (%)	No (%)	Yes (%)
**CT recommended by SNC guidelines**	No	89 (18)	35 (67)	54 (12)	37 (29)	17 (5)
	Yes	292 (58)	11 (21)	281 (62)	49 (38)	232 (71)
	Yes (since S100B was not analysed)	124 (25)	6 (12)	118 (26)	42 (33)	76 (23)
**Total**		**505**	**52**	**453**	**128**	**325**

Numbers in parentheses represent the percentage of the total number of each column.

SNC – Scandinavian Neurotrauma Committee Guidelines, S100B – Calcium-binding protein B, CT – Computed Tomography.

S100B levels were analysed in a total of 30 cases, compared with 136 analyses suggested by the guidelines. Among the analysed cases, 7 (23%) had concentrations below 0.10 μg/L, which is considered non-pathological according to SNC guidelines. In 127 cases, S100B was not measured despite guideline recommendations, meaning 9 cases were analysed in accordance with guidelines. Assuming the same 23% distribution of negative cases, an estimated 29 additional cases would likely have had non-pathological S100B levels. 54 cases underwent CT scans against guideline recommendations, see Table 2. If we include the 29 hypothetical negative S100B cases, this number rises to 83 cases. Following this hypothetical scenario, adherence to the SNC guidelines could lead to a potential reduction in the total number of CT scans of 18% (83 cases).

**Fig. 2:**
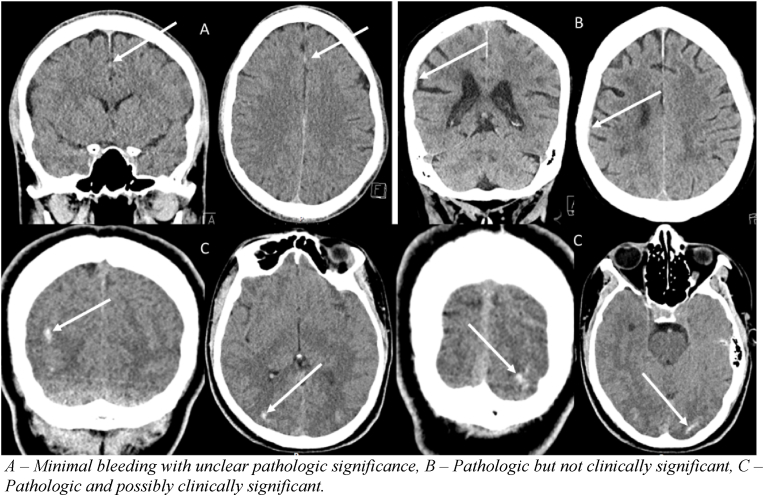
– Composite picture of three false negative cases, with three different degrees of significance. A – Minimal bleeding with unclear pathologic significance, B – Pathologic but not clinically significant, C – Pathologic and possibly clinically significant.

17 of 325 positive CT cases were false negatives, indicating pathological findings on CT despite SNC guidelines not recommending a CT. In one of these cases, S100B was analysed, despite the guidelines not recommending it. The result was >0,1 μg/L, suggesting a CT scan was warranted. However, since the guidelines recommended against analysing S100B, the case was considered a false negative. An additional review of the false negative cases by a senior neurosurgeon provided the following interpretation: Six cases had minimal bleeding if any, and the findings were unclear (category A). Seven were pathological but not clinically significant (category B). Lastly, four were deemed pathological with possible clinical significance (category C), one of which required neurosurgical intervention and one had a follow-up CT after two weeks, which showed resorption of the intracranial hematoma. Fig. 2 shows a characteristic CT image from each of the three categories. The distribution of sex, age, and injury type is outlined in Table 3. Notably, no statistical difference between these cases and the sample as a whole was found. The case which required neurosurgical intervention presented to the emergency department following a fall, in which the patient hit his head. Our review of the patient record resulted in the case being classified as minimal, according to the SNC guidelines, as no findings that would warrant a CT scan were described. However, in reality a CT scan was performed. The examination revealed a bilateral subdural hematoma (SDH) with mass effect, which required acute surgery. During the surgery a durotomy was performed and the surgeon noted an older chronic SDH. Retrospective review of the CT scan corroborated the assessment of chronic SDH. It should be noted that S100B was never analysed in this patient.

**Table 3:** Characteristics of the 17 cases of false negatives.

		n (%)	p-value^a^
**Sex**	Female	9 (53)	
	Male	8 (47)	
	Total	17	0.325
**Age group**	<30	2 (12)	
	31–40	0	
	41–50	1 (6)	
	51–60	4 (24)	
	61–70	2 (12)	
	71–80	5 (29)	
	81–90	1 (6)	
	>90	2 (12)	
	Age median	74 (q1/q3 – 55/84)	0.358
**CT findings (type of hematoma)**	SDH	9 (43)	
	SAH	9 (43)	
	Contusion	1 (5)	
	Parenchymal	2 (10)	
	Total^b^	21	
**Significance of finding**s^c^	Pathology unclear, not clinically significant	7 (41)	
	Pathologic but not clinically significant	6 (35)	
	Clinically significant	4 (24)	
	Total^b^	17	

a p-value of difference compared to the sample as a whole.

b Some patients presented with multiple bleedings.

c As interpreted by retrospective review of senior neurosurgeon. Q1/Q3 – 1st/3rd quartile, SDH – subdural hematoma, SAH – subarachnoid hematoma.

The results show that adherence to guidelines in the patient sample analysed was 56% during period of interest. As seen in Table 4, the main factor for non-adherence was S100B not being analysed, despite the guideline's recommendations. After accounting for confounding factors in binary logistic regression analysis, only the severity of TBI (SNC category) remained statistically significant (p < 0.001) with a correlation coefficient of 1.107.

**Table 4:** Showing adherence to guidelines and reasons for non-adherence in numbers and percentages.

Adherence to guidelines		Reasons for non-adherence	
Yes (%)	No (%)		n (%)
283 (56)	222 (44)	S100B was not analysed	126 (56)
		CT performed against recommendation	54 (24)
		S100B analysed against recommendation	18 (8)
		Not admitted for observation	11 (5)
		Other^a^	17 (8)
		**Total**	**226**^b^

a CT not performed, too short observation time, admission despite recommendation to discharge.

b Some cases had multiple reasons for non-adherence. SNC – Scandinavian Neurotrauma Committee, S100B – Calcium-binding protein B.

## 5 Discussion

The aim of this study was to validate the SNC guidelines in RÖC in a real-life situation, where a clinical emergency department and two city hospitals cover the acute care of a region of 310 000 inhabitants. It was hypothesised that the guidelines would be sensitive, and, by extension, an increased adherence would lead to a reduced number of CT scans being performed. In the present cohort, the sensitivity of the SNC guidelines was measured at 95%, however, adherence was measured at only 56%. The groups with the lowest adherence (SNC categories mild (low-risk) and minimal) were consistent with previous research (Ananthaharan et al., 2018a; Akre and Ingebrigtsen, 2021; Minkkinen et al., 2019; Faisal et al., 2023). Overall adherence was lower compared to some studies, which showed adherence of 63% (Ananthaharan et al., 2018a), 64% (Akre and Ingebrigtsen, 2021) and 80,7% (Minkkinen et al., 2019) respectively. Simultaneously, the results align with findings from other studies where adherence was found to be 55% (Faisal et al., 2023). A key difference in methodology in regards to the studies that showed higher adherence was how S100B was considered. One study defined compliance to the guidelines in regards to CT scan, not taking S100B alone into account (Akre and Ingebrigtsen, 2021). The other tested S100B in all patients included in the study, in order to have the analysis available in all cases providing more data when running analysis. It was concluded that none of the patients with a S100B level <0.10 μg/L had a positive CT scan (Minkkinen et al., 2019). Seeing as the main factor for non-adherence in the present study was the underuse of S100B, this may explain the difference in adherence. The study with similar adherence to guidelines as the present study treated S100B in a similar way, meaning that adherence was measured by correct use of both CT scans and S100B (Faisal et al., 2023). In this study, the main reason for non-adherence to guidelines was the underuse of S100B. This contrasts with findings from other studies, where the main issue is the overuse of CT scans (Akre and Ingebrigtsen, 2021; Minkkinen et al., 2019; Heskestad et al., 2012). It is possible that this difference is in part due to the chosen inclusion criteria, namely ICD-code S06, resulting in a higher number of cases where CT was warranted. Another factor to consider is that S100B no longer is part of any routine blood sampling at the emergency department in RÖC, but must be ordered from the physician. Seeing how some time has passed since the implementation of the guidelines and the criteria for when S100B is warranted is rather strict, it is possible that the knowledge regarding S100B has diminished over time. The data suggests that, despite the comprehensive nature of the SNC guidelines, their implementation is relatively low, in part due to the lack to S100B analysis. This should be considered when revising and re-implementing the guidelines in the future.

The results further show that the sensitivity of the SNC guidelines is in line with other validation studies, while specificity is slightly lower (Akre and Ingebrigtsen, 2021; Minkkinen et al., 2019; Undén et al., 2015). However, both the positive and negative predictive values differed greatly, with the PPV being higher and the NPV being lower than in other studies (Minkkinen et al., 2019; Undén et al., 2015). This difference may be explained, in large part, by the chosen inclusion criteria for this study, namely in the choice of ICD coding. As mentioned above, by solely extracting patients coupled to the ICD code S06, it may have led to a higher number of cases with positive CT results compared to other studies and methods. This would explain the higher PPV in combination with a comparable sensitivity as other studies. The higher number of positive CT results does not directly relate to the lower specificity and lower NPV. However, if the proportion of positive and negative CT results is skewed in favour of positive, this would affect NPV and specificity as well.

The sensitivity and PPV show that the SNC guidelines accurately predict which patients are at risk for CT-positive TBI. However, it is noteworthy that 17 cases were false negatives and that the NPV was lower than in other studies (Minkkinen et al., 2019; Undén et al., 2015). One of these cases also underwent emergency surgery. This number of false negatives, and especially the case requiring surgery, are rare findings in the history of validation studies assessing the performance of S100B and the SNC guidelines. Since the surgery revealed a chronic SDH, it could be considered an accidental finding rather than a pathology related to the TBI. However, if the patient had been managed per any of the other guidelines outlined in Appendix A*,* a CT scan would have been performed due to the patient's age. Should age be considered in future revisions of the SNC guidelines, 65 years of age would align with several of the contemporary guidelines (Smits et al., 2007; Stiell et al., 2001; Haydel and Blaudeau, 2000; National Institute for Health and Care Excellence).

The age distribution of the included sample indicates that mild and moderate TBI are more common in the elderly (≥70 years) and middle-aged adults (31–40 years). This is partly in line with previous research, which states that the elderly are at higher risk of TBI (Garza et al., 2020; Thompson et al., 2006). On the other hand, the Centers for Disease Control and Prevention (CDC) cite young adults (15–24 years) as having the second-highest risk, excluding children (Centers for Disease Control and Prevention). Concussion is the most common type of injury in adolescents (15–19 years) and young adults (20–24 years) (Zhang et al., 2016). The sole use of the ICD code S06 for the purpose of this study may, in part, explain the deviation from previous observations. Furthermore, this study confirms earlier observations that both young age and male sex is associated with more severe TBI and higher incidence of hospital admission and/or active treatment, an observation contributed partly to riskier behaviour (Mikolić et al., 2021; Mikolic et al., 2021; Tamás et al., 2019).

However, several limitations need to be addressed. As previously mentioned, the sole use of the ICD code S06 (intracranial injury) is likely to have skewed the patient sample towards those with pathological findings, mostly intracranial haemorrhage, which also makes it likely that many patients with minimal TBI were not included in the sample. The combination of S06 and S09.9 would likely have resulted in a more accurate patient sample with minimal TBI and/or normal CT scans. The decision to only include S06 was based on focusing resources on more relevant mild TBI cases, omitting minimal/negligible TBI.

The SNC guidelines rely heavily on the GCS scoring system. The RLS scale is however more commonly used in Sweden, in both prehospital and emergency room settings. The cases with only RLS noted in the records as consciousness scale were not excluded from the study, but were instead converted to GCS scoring system. Since no conventional conversion guide exists, this may have resulted in misclassification of some cases despite the conversion method used being previously employed with satisfactory results (Eric Thelin et al., 2023; Vestlund et al., 2022). On the other hand, as RLS is the most commonly used scale, not including patients with only this scale documented would not reflect a realistic clinical Swedish setting. This observation, however, could pose as an argument to align with international standards and implement the GCS-scale, in coherence with SNC guidelines, as the RLS scale is not as well investigated as a prognostic tool (Reith et al., 2016). Including other regions would have increased the external validity of the study. This will be considered as a next step, following the methodological experiences gained through the present study.

The results of this study show that the sensitivity of the SNC guidelines was high, as previously reported in the literature (Akre and Ingebrigtsen, 2021; Minkkinen et al., 2019; Faisal et al., 2023; Undén et al., 2015; Ananthaharan et al., 2018b), while adherence was lower than or equal to similar studies with comparable settings (Akre and Ingebrigtsen, 2021; Minkkinen et al., 2019; Faisal et al., 2023; Ananthaharan et al., 2018b). The finding of a chronic SDH requiring surgical intervention, missed under current definitions of SNC guidelines, raises the question of whether age alone should be included in the SNC guidelines, as it is in other CT-based decision guides. A Finnish study makes similar observations, where two false negative cases where identified, both of which were elderly patients (Minkkinen et al., 2019).

## 6 Conclusions

The sensitivity of the SNC guidelines was high and measured at 95%. Adherence to the SNC guidelines in RÖC was 56%, indicating a need for a re-evaluation of TBI care in the emergency department. The SNC has exhibited the ability to screen for clinically significant CT-positive pathologies, both in the current study and its predecessors. Increased adherence can lead to a further reduction in the number of unnecessary CT scans by an estimated 18%. Additional studies, including a wider range of ICD codes, are needed to further evaluate the SNC guidelines, especially concerning age as a criterion for CT scan.

## Data availability

The data that support the findings of this study are available from the corresponding author upon reasonable request.

## Funding statement

This research was funded by the Swedish Brain Foundation (FO2024-0040), the Swedish Research Council (2023-02044), and Promobilia foundation (A24289) research grants.

## Declaration of competing interest

The authors declare that they have no known competing financial interests or personal relationships that could have appeared to influence the work reported in this paper.

## Figures and Tables

**Table:** 

	SNC guidelines (Undén et al., 2013)	CHIPPR (Smits et al., 2007)	CCTHR (Stiell et al., 2001)	NOHCTR (Haydel and Blaudeau, 2000)	NICE guidelines (National Institute for Health and Care Excellence)
**When to use**	Adult patients within 24 h of head injury and GCS 9-15	Patients ≥16 years old within 24 h of trauma and GCS 13-15	Patients with GCS 13–15 and at least one of the following: Loss of consciousness Amnesia to the head injury event Witnessed disorientation	Patients with head injury and loss of consciousness with GCS 15 and normal neurologic status	Patients ≥16 years old who have sustained a head injury
**Exclusion criteria**			Age <16, blood thinners, or seizure after injury		
**Criteria which alone warrants CT scan**	GCS 9-13 Posttraumatic seizures Focal neurological deficit Signs of depressed or skull base fracture Shunt-treated hydrocephalus Anticoagulants or coagulation disorder Age ≥65 AND anti-platelet medication S100B ≥ 0.10	Age ≥60 GCS <15 GCS deterioration ≥2 point within 1 h Injury suggesting skull fracture Post-traumatic amnesia >4 h Dangerous mechanism^a^ Vomiting Use of anticoagulants Post-traumatic seizures	Age ≥65 GCS <15 at 2 h post-injury Suspected open or depressed skull fracture Signs of basal skull fracture Vomiting ≥2 episodes Retrograde amnesia to the event >30 min Dangerous mechanism^a^	Age ≥60 Headache Vomiting Alcohol or drug intoxication Anterograde amnesia Visible trauma above the clavicle Seizures	GCS <15 at 2 h post-injury GCS <12 on initial assessment Suspected open or depressed skull Signs of basal skull fracture Post-traumatic seizure Focal neurological deficit Vomiting ≥2 episodes
**Criteria which in combination with others warrants CT scan**	Suspected/confirmed loss of consciousness Vomiting ≥2 episodes GCS 14 and no other symptoms	Age 40-60 GCS deterioration 1 points within 1 h Skull contusion Post traumatic amnesia 2–4 h Anterograde amnesia Fall from any elevation Neurological deficit Loss of consciousness			Loss of consciousness or amnesia in combination with any of the following: Age ≥65 Any current bleeding or clotting disorders Dangerous mechanism of injury Retrograde amnesia to the event >30 min

Overview of contemporary guidelines for deciding on CT scan in patients with head injury. ^1^ Pedestrian struck by motor vehicle, occupant ejected from motor vehicle, or fall from >3 feet or >5 stairs. SNC – Scandinavian Neurotrauma Committee, CHIPPR – CT in Head Injury Patients Prediction Rule, NOHCTR – New Orleans Head CT Rule, CCTHR – Canadian CT Head Rule, NICE – the National Institute for Health and Care Excellence, GCS – Glasgow Coma Scale, S100B – calcium binding protein B.
